# The Recombination Landscape in Wild House Mice Inferred Using Population Genomic Data

**DOI:** 10.1534/genetics.117.300063

**Published:** 2017-07-26

**Authors:** Tom R. Booker, Rob W. Ness, Peter D. Keightley

**Affiliations:** * Institute of Evolutionary Biology, University of Edinburgh, EH9 3FL, United Kingdom; † Department of Biology, University of Toronto Mississauga, Ontario, L5L 1C6, Canada

**Keywords:** *Mus musculus*, recombination, wild Mice, population genomics

## Abstract

Characterizing variation in the rate of recombination across the genome is important for understanding several evolutionary processes. Previous analysis of the recombination landscape in laboratory mice has revealed that the different subspecies have different suites of recombination hotspots. It is unknown, however, whether hotspots identified in laboratory strains reflect the hotspot diversity of natural populations or whether broad-scale variation in the rate of recombination is conserved between subspecies. In this study, we constructed fine-scale recombination rate maps for a natural population of the Eastern house mouse, *Mus musculus castaneus*. We performed simulations to assess the accuracy of recombination rate inference in the presence of phase errors, and we used a novel approach to quantify phase error. The spatial distribution of recombination events is strongly positively correlated between our *castaneus* map, and a map constructed using inbred lines derived predominantly from *M. m. domesticus*. Recombination hotspots in wild *castaneus* show little overlap, however, with the locations of double-strand breaks in wild-derived house mouse strains. Finally, we also find that genetic diversity in *M. m. castaneus* is positively correlated with the rate of recombination, consistent with pervasive natural selection operating in the genome. Our study suggests that recombination rate variation is conserved at broad scales between house mouse subspecies, but it is not strongly conserved at fine scales.

IN many species, crossing-over events are not uniformly distributed across chromosomes. Understanding this variation and its causes is important for many aspects of molecular evolution. Experiments in laboratory strains or managed populations that examine the inheritance of markers through pedigrees have produced direct estimates of crossing-over rates in different genomic regions. Studies of this kind are impractical for many wild populations, however, because pedigrees are largely unknown (but see Johnston *et al.* 2016). In mice, there have been several genetic maps published (*e.g.*, Jensen-Seaman *et al.* 2004; Paigen *et al.* 2008; Cox *et al.* 2009; Liu *et al.* 2014), typically using the classical inbred laboratory strains, which are predominantly derived from the Western European house mouse subspecies, *Mus musculus domesticus* (Yang *et al.* 2011). Recombination rate variation in laboratory strains may not, therefore, reflect rates and patterns in wild mice of other subspecies. In addition, recombination rate modifiers may have become fixed in the process of laboratory strain management. On the other hand, directly estimating recombination rates in wild house mice is not feasible without both a population’s pedigree and many genotyped individuals (but see Wang *et al.* 2017).

Patterns of linkage disequilibrium (LD) in a sample of individuals drawn from a population can be used to infer variation in the rate of recombination across the genome. Coalescent-based methods have been developed to indirectly estimate recombination rates at very fine scales (Hudson 2001; McVean *et al.* 2002, 2004; Auton and McVean 2007; Chan *et al.* 2012). Recombination rates estimated in this way reflect long-term variation in crossing-over in the population’s history, and are averages between the sexes. Methods using LD have been applied to explore variation in recombination rates among mammals and other eukaryotes, and have demonstrated that recombination hotspots are associated with specific genomic features (Myers *et al.* 2010; Paigen and Petkov 2010; Singhal *et al.* 2015).

The underlying mechanisms explaining the locations of recombination events have been the focus of much research. In house mice and in most other mammals, the *PRDM9* zinc-finger protein binds to specific DNA motifs, resulting in an increased probability of double-strand breaks (DSBs), which can then be resolved by reciprocal crossing-over or gene conversion (Grey *et al.* 2011; Baudat *et al.* 2013). Accordingly, it has been shown that recombination hotspots are enriched for *PRDM9* binding sites (Myers *et al.* 2010; Brunschwig *et al.* 2012). *PRDM9*-knockout mice still exhibit hotspots, but in dramatically different genomic regions (Brick *et al.* 2012). Variation in *PRDM9*, specifically in the exon encoding the zinc-finger array, results in different binding motifs (Baudat *et al.* 2010). Davies *et al.* (2016) generated a line of mice in which the exon encoding the portion of the *PRDM9* protein specifying the DNA binding motif was replaced with the orthologous human sequence. The recombination hotspots they observed in this “humanized” line of mice were enriched for the human *PRDM9* binding motif.

Great ape species each have different *PRDM9* alleles (Schwartz *et al.* 2014) and relatively little hotspot sharing (Winckler *et al.* 2005; Stevison *et al.* 2016). The broad-scale recombination landscapes of the great apes are, however, strongly positively correlated (Stevison *et al.* 2011, 2016), suggesting that hotspots evolve rapidly, but that the overall genetic map changes more slowly. Indeed, broad-scale recombination rates are positively correlated between closely related species pairs with different hotspot locations (Smukowski and Noor 2011), and between species that share hotspots or lack them altogether (Singhal *et al.* 2015; Smukowski Heil *et al.* 2015).

It has been suggested that a population ancestral to the *M. musculus* subspecies complex split into the present-day subspecies ∼350,000 years ago (Geraldes *et al.* 2011). In this time, functionally distinct *PRDM9* alleles and distinct suites of hotspots evolved in the different subspecies (Smagulova *et al.* 2016). In addition, there is variation in the recombination rate at relatively broad scales across several regions of the genome between members of the *M. musculus* subspecies complex (Dumont *et al.* 2011), and recombination rates vary between recently diverged *M. m. domesticus* populations (Wang *et al.* 2017). Brunschwig *et al.* (2012) analyzed single nucleotide polymorphism (SNP) data for classical laboratory strains of mice and used an LD-based approach to estimate the sex-averaged recombination landscape for the 19 autosomes. Their genetic map is similar to a genetic map generated using crosses by Cox *et al.* (2009). However, both studies were conducted using inbred lines whose ancestry is largely *M. m. domesticus* (Yang *et al.* 2011), so their recombination landscapes may be different from other members of the *M. musculus* subspecies complex.

In this study, we constructed genetic maps for the house mouse subspecies *M. m. castaneus*. We used the genome sequences of 10 wild-caught individuals of *M. m. castaneus* from the species’ assumed ancestral range, originally reported by Halligan *et al.* (2013). In our analysis, we first phased SNPs and estimated rates of error in phasing. Second, we simulated data to assess the power of estimating recombination rates based on only 10 individuals, and the extent by which phase errors lead to biased estimates of the rate of recombination. Finally, using an LD-based approach, we inferred a sex-averaged genetic map and compared this to previously published maps for *M. musculus*. We show that broad-scale variation in recombination rates in *M. m. castaneus* is similar to that seen in the classical inbred strains. However, we show that the locations of potential recombination hotspots in *M. m. castaneus* exhibit little overlap with those reported in wild-derived laboratory strains.

## Materials and Methods

### Polymorphism data for *Mus musculus* *castaneus*

We analyzed the genome sequences of 10 wild-caught *M. m. castaneus* individuals (Halligan *et al.* 2013). Samples were from North-West India, a region that is believed to be within the ancestral range of the house mouse. Mice from this region have the highest genetic diversity among the *M. musculus* subspecies (Baines and Harr 2007). In addition, the individuals sequenced showed little evidence for substantial inbreeding, and a population structure analysis suggested that they represent a single population (Halligan *et al.* 2010). Halligan *et al.* (2013) sequenced individual genomes to high coverage using multiple libraries of Illumina paired-end reads, and mapped these to the mm9 reference genome using BWA (Li and Durbin 2009). Mean coverage was >20× and the proportion of the genome with >10× coverage was >80% for all individuals sampled (Halligan *et al.* 2013). Variants were called with the Samtools *mpileup* function (Li *et al.* 2009) using an allele frequency spectrum (AFS) prior. The AFS was obtained by iteratively calling variants until the spectrum converged. After the first iteration, all SNPs at frequencies >0.5 were swapped into the mm9 genome to construct a reference genome for *M. m. castaneus*, which was used for subsequent variant calling (for further details see Halligan *et al.* 2013). The variant call format (VCF) files generated by Halligan *et al.* (2013) were used in this study. In addition, alignments of *Mus famulus* and *Rattus norvegicus* to the mm9 genome, also generated by Halligan *et al.* (2013), were used as outgroups.

For the purpose of estimating recombination rates, variable sites were filtered on the basis of the following conditions. Insertion/deletion polymorphisms were excluded, because the method used to phase variants cannot process these sites. Sites at which more than two alleles segregated and those that failed the Samtools Hardy-Weinberg equilibrium test (*P* < 0.002) were also excluded. The hypermutability of CpG sites violates the assumption of a single mutation rate. We defined sites as CpG-prone if they were preceded by a C, or followed by a G, in *M. m. castaneus*, *M. famulus* or *R. norvegicus*.

### Inferring phase and estimating switch error rates

LDhelmet estimates recombination rates from a sample of phased chromosomes or haplotypes drawn from a population. To infer haplotypes, heterozygous SNPs called in *M. m. castaneus* were phased using read-aware phasing in ShapeIt2 (Delaneau *et al.* 2013), which phases variants at the level of whole chromosomes using sequencing reads that span multiple heterozygous sites (phase-informative reads, PIRs), and LD. Incorrectly phased heterozygous sites, termed switch errors, tend to upwardly bias estimates of the recombination rate, because they appear identical to legitimate crossing-over events. To assess the impact of incorrect phasing on recombination rate inference, we quantified the switch error rate as follows. The sample of *M. m. castaneus* comprised seven females and three males. The X-chromosome variants in males therefore represent perfectly phased haplotypes. We merged the BAM alignments of short reads for the X-chromosomes of the three males (samples H12, H28, and H34 from Halligan *et al.* 2013) to make three datasets of pseudofemales where the true haplotypes are known (H12 + H28 = H40; H12 + H34 = H46; H28 + H34 = H62). We then jointly recalled variants in the seven female samples plus the three pseudofemales using an identical pipeline as Halligan *et al.* (2013), using the same AFS prior.

Switch error rates in Shapeit2 are sensitive both to coverage and quality (per genotype and per variant) (Delaneau *et al.* 2013). We explored the effects of different filter parameters on switch error rates using the X-chromosomes of the pseudofemales. We filtered SNPs based on combinations of variant and genotype quality scores (QUAL and GQ, respectively) and on an individual’s sequencing depth (DP) (Supplemental Material, Table S1). For the individual-specific statistics (DP and GQ), if a single individual failed a particular filter, then that SNP was excluded from further analyses. By comparing the known X-chromosome haplotypes and those inferred by ShapeIt2, we calculated switch error rates as the ratio of incorrectly resolved heterozygous SNPs to the total number of heterozygous SNPs for each pseudofemale individual. We used these results to apply filter parameters to the autosomal data that generated a low switch error rate, while maintaining a high number of heterozygous SNPs. We obtained 20 phased haplotypes for each of the 19 mouse autosomes, and 14 for the X-chromosome (plus the three from the male samples). With these, we estimated the recombination rate landscape for *M. m. castaneus*.

### Estimating genetic maps and validation of the approach

LDhelmet (v1.7; Chan *et al.* 2012) generates a sex-averaged genetic map from a sample of haplotypes assumed to be drawn from a randomly mating population. Briefly, LDhelmet examines patterns of LD in a sample of phased chromosomal regions and uses a composite likelihood approach to infer recombination rates between adjacent SNPs. LDhelmet appears to perform well for species of large effective population size (*N_e_*) and has been shown to be robust to the effects of selective sweeps, which appear to reduce diversity in and around functional elements of the *M. m. castaneus* genome (Halligan *et al.* 2013). The analyses of Chan *et al.* (2012), in which the software was tested, were performed with a larger number of haplotypes than we have in our sample. To assess whether our smaller sample size still gives reliable genetic maps, we validated and parameterized LDhelmet using simulated datasets (see below). It should be noted, however, that model underlying LDhelmet assumes recombination-drift equilibrium. Violation of this assumption may therefore result in biased recombination rate estimates.

A key parameter in LDhelmet is the block penalty, which determines the extent by which likelihood is penalized by spatial variation in the recombination rate, such that a high block penalty results in a smoother recombination map. We performed simulations to determine the block penalty that produces the most accurate estimates of the recombination rate in chromosomes that have diversity and base content similar to *M. m. castaneus*. Chromosomes with constant values of *ρ* (*4N_e_r*) ranging from 2 × 10^−6^ to 2 × 10^1^ were simulated in SLiM v1.8 (Messer 2013). For each value of *ρ*, 0.5 Mbp of neutrally evolving sequence was simulated for populations of *N* = 1000 diploid individuals. Mutation rates in the simulations were set using the compound parameter *θ* = *4N_e_μ*, where *μ* is the per-base, per-generation mutation rate. The mutation and recombination rates of the simulations were scaled to *θ*/*4N* and *ρ*/*4N*, respectively. *θ* was set to 0.01 in the simulations, because this value is close to the genome-wide average for our data, based on pairwise differences. Simulations were run for 10,000 generations in order to achieve equilibrium diversity, at which time 10 diploid individuals were sampled. Each simulation was repeated 20 times, resulting in 10 Mbp of sequence for each value of *ρ*. The SLiM output files were converted to sequence data suitable for analysis by LDhelmet using a custom Python script that incorporated the mutation rate matrix estimated for non-CpG prone sites in *M. m. castaneus* (see below). Following (Chan *et al.* 2012), we inferred recombination rates from the simulated data in windows of 4400 SNPs with a 200 SNP overlap between windows. We analyzed the simulated data using LDhelmet with block penalties of 10, 25, 50, and 100. The default parameters of LDhelmet are tuned to analyze *Drosophila melanogaster* data (Chan *et al.* 2012). Since the *D. melanogaster* population studied by Chan *et al.* (2012) has comparable nucleotide diversity to *M. m. castaneus*, we used default values for other parameters, with the exception of the block penalty.

Errors in phase inference, discussed above, may bias our estimates of the recombination rate, since they appear to break apart patterns of LD. We assessed the impact of these errors on recombination rate inference by incorporating them into the simulated data at a rate estimated from the pseudofemale individuals. For each of the 10 individuals drawn from the simulated populations, switch errors were randomly introduced at heterozygous positions at the rate estimated using the SNP filter set chosen on the basis of the pseudofemale analysis (see *Results*). We then inferred recombination rates for the simulated population using these error-prone data, as above. We assessed the effect of switch errors on recombination rate inference by comparing estimates from the simulated data with and without switch errors. It is worth noting that switch errors may undo crossing-over events, and thereby reduce inferred recombination rates if they affect heterozygous SNPs located at recombination breakpoints.

### Recombination rate estimation for *M. m. castaneus*

We used LDhelmet (Chan *et al.* 2012) to estimate recombination rate landscapes for each of the *M. m. castaneus* autosomes and the X-chromosome. A drawback of LD-based approaches is that they estimate sex-averaged recombination rates. This is a limitation of our study as there are known differences in recombination rates between the sexes in *M. musculus* (Cox *et al.* 2009; Liu *et al.* 2014).

We used *M. famulus* and *R. norvegicus* as outgroups to assign ancestral states for polymorphic sites. LDhelmet incorporates the mutation matrix and a prior probability on the ancestral allele at each variable position as parameters in the model. We obtained these parameters as follows. For non-CpG prone polymorphic sites, if the two outgroups shared the same allele, we assigned that allele as ancestral, and such sites were then used to populate the mutation matrix (Chan *et al.* 2012). This approach ignores the possibility of back mutation and homoplasy. To account for this uncertainty, LDhelmet incorporates a prior probability on the ancestral base. Following Singhal *et al.* (2015), at resolvable sites (*i.e.*, where both outgroups agreed) the ancestral base was given a prior probability of 0.91, with 0.03 assigned to each of the three remaining bases. This was done to provide high confidence in the ancestral allele, but also to include the possibility of ancestral allele misinference. At unresolved sites (*i.e.*, if the outgroups disagreed or there were alignment gaps in either outgroup), we used the stationary distribution of allele frequencies from the mutation rate matrix as the prior (Table S2).

We analyzed a total of 44,835,801 SNPs in LDhelmet to construct genetic maps for the *M. m. castaneus* autosomes and the X-chromosome. Following Chan *et al.* (2012), windows of 4400 SNPs, overlapping by 200 SNPs on either side were analyzed. We ran LDhelmet for a total of 1,000,000 iterations, discarding the first 100,000 as burn-in. A block penalty of 100 was chosen to obtain conservatively estimated broad-scale genetic maps. For the purposes of identifying recombination hotspots, we reran the LDhelmet analysis with a block penalty of 10. We analyzed all sites that passed the filters chosen using the pseudofemale phasing analysis regardless of CpG status; note that excluding CpG-prone sites removes ∼50% of the available data, and thus would substantially reduce the power to infer recombination rates. We assumed *θ* = 0.01, the approximate genome-wide level of neutral diversity in *M. m. castaneus*, and included ancestral allele priors and the mutation rate matrix for non-CpG sites as parameters in the model. Following the analyses, we removed overlapping SNPs and concatenated SNP windows to obtain recombination maps for whole chromosomes.

It is worthwhile noting that our genetic maps were constructed with genotype calls made using the mm9 version of the mouse reference genome. This version was released in 2007 and there have been subsequent versions released since then. However, previously published genetic maps for *M. musculus* were constructed using mm9, so we used that reference to make comparisons (see below).

### Broad-scale comparison to previously published maps

We compared the *M. m. castaneus* genetic map inferred using a block penalty of 100 with two previously published maps for *M. musculus*. The first map was generated by analyzing the inheritance patterns of markers in crosses between inbred lines (Cox *et al.* 2009) (downloaded from http://cgd.jax.org/mousemapconverter/). We refer to this map as the Cox map. The second map was generated by Brunschwig *et al.* (2012) by analyzing SNPs in classical inbred mouse lines using LDhat (Auton and McVean 2007), the software upon which LDhelmet is based (available at http://www.genetics.org/content/early/2012/05/04/genetics.112.141036). We refer to this map as the Brunschwig map. The Cox and Brunschwig maps were constructed using far fewer markers than the present study, *i.e.*, ∼500,000 and ∼10,000 SNPs, respectively, compared to the ∼45,000,000 used to generate ours. Recombination rate variation in the Cox and Brunschwig maps likely reflects that of *M. m. domesticus*, since both were generated using classical strains of laboratory mice, which are predominantly of *M. m. domesticus* origin (Yang *et al.* 2011). For example, in the classical inbred strains analyzed by Cox *et al.* (2009), the mean genome-wide ancestry attributable to *M. m. domesticus*, *M. m. musculus* and *M. m. castaneus* are 94.8, 5.0, and 0.2%, respectively [data downloaded from the Mouse Phylogeny Viewer (Wang *et al.* 2012) http://msub.csbio.unc.edu]. The ancestry proportions for all classical strains, 60 of which were analyzed by Brunschwig *et al.* (2012), are similar (Yang *et al.* 2011).

Recombination rates in the Brunschwig map and our *castaneus* map were estimated in units of *ρ* = *4N_e_r*. For comparison purposes, we converted these units to centimorgans per megabase using frequency-weighted means, as follows. LDhat and LDhelmet provide estimates of *ρ* (per kilobase pair and base pair, respectively) between pairs of adjacent SNPs. For each chromosome, we calculated cumulative *ρ*, while accounting for differences in the physical distance between adjacent SNPs by using the number of bases separating a pair of SNPs to weight that pair’s contribution to the total. By setting the total map length for each chromosome to that of Cox *et al.* (2009), we converted the cumulative *ρ* at each analyzed SNP position to centimorgan values.

At the level of whole chromosomes, we compared mean recombination rate estimates for *castaneus* with several previously published maps. Frequency-weighted mean recombination rates (in terms of *ρ*) for each chromosome in the *castaneus* and Brunschwig maps were compared with centimorgans per megabase values obtained by Cox *et al.* (2009), and with independent estimates of per chromosome recombination rates (Jensen-Seaman *et al.* 2004). Pearson correlations were calculated for each comparison.

At the megabase pair scale, we compared variation in recombination rates across the autosomes in the different maps using windows of varying length. We calculated Pearson correlations between the frequency weighted-mean recombination rates (in centimorgans per megabase) in nonoverlapping windows of 1–20 Mbp for the *castaneus*, Cox and Brunschwig maps. For visual comparison of the *castaneus* and Cox maps, we plotted recombination rates in sliding windows of 10 Mbp, offset by 1 Mb.

### Fine-scale recombination rate variation

To assess the distribution of recombination events in *M. m. castaneus* on a fine scale, we used Gini coefficients and Lorenz curves as quantitative measures of the extent of heterogeneity (*e.g.*, Kaur and Rockman 2014). In the context of a genetic map, Gini coefficients close to zero represent more uniform distributions of crossing-over rates, whereas values closer to one indicate that recombination events are restricted to a small number of locations. We analyzed genetic maps generated using a block penalty of 10 to construct Lorenz curves and calculated their Gini coefficients for each chromosome separately.

Recombination hotspots can be operationally defined as small windows of the genome that exhibit elevated rates of recombination relative to surrounding regions. To estimate the locations of potential recombination hotspots, we adapted a script used by Singhal *et al.* (2015). We divided the genome into nonoverlapping windows of 2 kbp, and, using the maps generated with a block penalty of 10, classified as putative hotspots all windows where the recombination rate was at least 5× greater than the recombination rate in the surrounding 80 kbp. Recombination hotspots may be >2 kbp, so neighboring analysis windows that exhibited elevated recombination rates were merged.

We investigated whether fine-scale recombination rate variation in wild-caught *M. m. castaneus* is similar to that reported for wild-derived inbred lines. Smagulova *et al.* (2016) generated sequencing reads corresponding to the locations of DSBs (hereafter DSB hotspots) in inbred strains of mice derived from each of the principal *M. musculus* subspecies and *M. m. molossinus*, an intersubspecific hybrid of *M. m. castaneus* and *M. m. musculus*. We used the overlap between our putative hotspots and their DSB hotspots for testing similarity. However, the coordinates of DSB hotspots were reported with respect to the mm10 genome (Smagulova *et al.* 2016). To allow comparisons with our putative hotspots, we converted the coordinates of DSB breaks in the mm10 reference to mm9 coordinates using the University of California Santa Cruz (UCSC) LiftOver tool (https://genome.ucsc.edu/cgi-bin/hgLiftOver), with default parameters. We compared the locations of putative hotspots identified in our *castaneus* map with the locations of DSB hotspots using BedTools v2.17.0 (Quinlan and Hall 2010) by counting the number that overlapped. To determine the number of overlaps expected to be seen by chance, we used a randomization approach as follows. The locations of our putative hotspots were randomized with respect to chromosome, and these shuffled coordinates were compared to the locations of DSB hotspots. For each of the inbred strains analyzed by Smagulova *et al.* (2016), this procedure was repeated 1000 times. The maximum number of overlapping DSB and putative *castaneus* hotspots observed across all 1000 replicates was taken as an ∼0.1% significance threshold.

### Examining the correlation between recombination rate and properties of protein-coding genes

We used our *castaneus* map to examine the relationship between recombination rates and nucleotide diversity and divergence as follows. We obtained the coordinates of the canonical spliceforms of protein coding genes, orthologous between mouse and rat from Ensembl Biomart (Ensembl Database 67; http://www.ensembl.org/info/website/archives/index.html). For each protein-coding gene, we calculated the frequency-weighted mean recombination rate from the broad-scale map. Using the approximate *castaneus* reference described above, along with the outgroup alignments, we obtained the locations of fourfold degenerate synonymous sites and current GC content for each gene. If a site was annotated as fourfold in all three species considered, it was used for further analysis. We removed poor quality alignments between mouse and rat that exhibited spurious excesses of mismatched sites, where >80% of sites were missing. We also excluded five genes where there were mismatches with the rat sequence at all non-CpG prone fourfold sites, since it is likely that these also represent incorrect alignments. After filtering, there were a total of 18,171 protein-coding genes for analysis.

We examined the correlation between the local recombination rate in protein-coding genes and nucleotide diversity, divergence from the rat and GC-content. Variation in the mutation rate across the genome is a potentially important confounding factor. For example, if the recombination rate and mutation rate are positively correlated, we would expect a positive correlation between neutral nucleotide diversity and recombination rate. Because of this, we also examined the correlation between the ratio of nucleotide diversity to divergence from *R. norvegicus* at putatively neutral sites and the rate of recombination. We calculated correlations for all sites and for non-CpG-prone sites only. We used nonparametric Kendall rank correlations for all comparisons.

Analyses were conducted using Python scripts, except for the correlation analyses, which were conducted using R (R Core Team 2016) and hotspot identification, which was done using a Python script adapted from one provided by Singhal *et al.* (2016).

### Data availability

The authors confirm that all data necessary for performing the analyses described in the article are fully described in the text. Recombination maps are available in a compressed form from https://github.com/TBooker/M.m.castaneus_recombination-maps.

## Results

### SNP phasing and estimating the switch error rate

To infer genetic maps using our sample of individuals, we required phased SNPs. Taking advantage of the high sequencing depth of the sample generated by Halligan *et al.* (2013), and using a total of 44,835,801 SNPs (Table S3), we phased SNPs using ShapeIt2, an approach that uses LD and sequencing reads to resolve haplotypes.

We quantified the switch error rate incurred when inferring phase by analyzing pseudofemale individuals. After filtering variants, ShapeIt2 returned low switch error rates for all parameter combinations tested (Table S1). We therefore applied a set of filters (GQ > 15, QUAL > 30) to apply to the actual data that predicted a mean switch error rate of 0.46% (Table S1). When applied to the actual data these filters removed 44% of the total number of called SNPs (Table S3). More stringent filtering resulted in slightly lower mean switch error rates, but also removed many more variants (Table S1), reducing our ability to estimate recombination rates at a fine scale.

### Simulations to validate the application of LDhelmet

**Figure 1:**
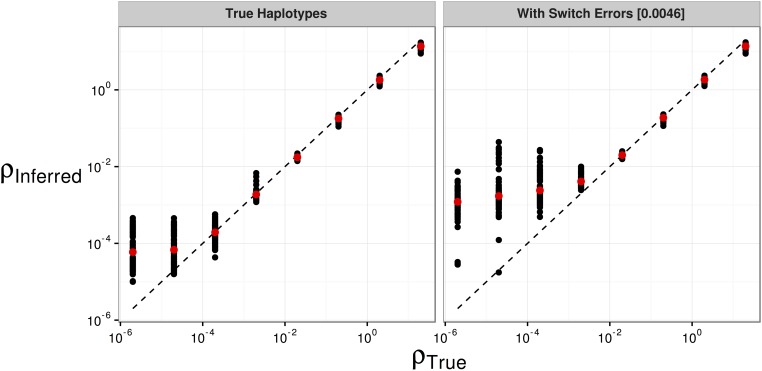
The effect of switch errors on the mean recombination rate inferred using LDhelmet with a block penalty of 100. Each black point represents results for a window of 4000 SNPs, with 200 SNPs overlapping between adjacent windows, using sequences simulated in SLiM for a constant value of ρ/bp. Red points are mean values. Switch errors were randomly incorporated at heterozygous SNPs with probability 0.0046. The dotted line shows the value when the inferred and true rates are equal.

We used simulations to assess the performance of LDhelmet when applied to our dataset. In the absence of switch errors, LDhelmet accurately inferred the average recombination rate down to values of *ρ*/bp = 2 × 10^−4^. Below this value, LDhelmet overestimated the scaled recombination rate (Figure 1). With switch errors incorporated into simulated data, LDhelmet accurately estimated *ρ*/bp in the range 2 × 10^−3^ to 2 × 10^2^. When the true *ρ*/bp was <2 × 10^−3^, however, LDhelmet overestimated the mean recombination rate for 0.5 Mbp regions (Figure 1). This behavior was consistent for all block penalties tested (Figure S1). We found that inferred rates of recombination typically fell within the range accurately estimated by LDhelmet (Figure S2 and Table 1).

**Table 1:** Summary of sex-averaged recombination rates estimated for the *M. m castaneus* autosomes compared with published rates

Chromosome	Cox*^a^* cM/Mb	*castaneus*		Brunschwig*^b^*	
		Freq. Weighted Mean	*N_e_* Estimate	Freq. Weighted Mean	*N_e_* Estimate
1	0.50	0.0079	395,000	0.000015	745
2	0.57	0.0088	386,000	0.000015	653
3	0.52	0.0083	400,000	0.000014	693
4	0.56	0.0091	408,000	0.000020	889
5	0.59	0.0090	382,000	0.000015	646
6	0.53	0.0089	421,000	0.000015	728
7	0.58	0.0100	429,000	0.000019	801
8	0.58	0.0094	404,000	0.000014	610
9	0.61	0.0096	394,000	0.000018	749
10	0.61	0.0096	392,000	0.000023	928
11	0.70	0.0102	365,000	0.000019	689
12	0.53	0.0089	420,000	0.000019	897
13	0.56	0.0095	426,000	0.000014	629
14	0.53	0.0084	395,000	0.000013	632
15	0.56	0.0083	371,000	0.000024	1080
16	0.59	0.0091	386,000	0.000017	721
17	0.65	0.0087	335,000	0.000052	2020
18	0.66	0.0098	371,000	0.000021	785
19	0.94	0.0122	323,000	0.000026	681
X	0.48	0.0026	137,000	—	—
Mean		0.0092		0.000020	

Rates for the *castaneus* and Brunschwig maps are presented in terms of 4*N_e_r*/bp. Estimates of *N_e_* were obtained by assuming the recombination rates from Cox *et al.* (2009).

a Cox *et al.* (2009)

b Brunschwig *et al.* (2012)

### Recombination rates in the *M. m. castaneus* genome

We constructed two maps of recombination rate variation for *M. m. castaneus* using LDhelmet. The first was a broad-scale map, constructed using a block penalty of 100 (hereafter referred to as the broad-scale map). For the second fine-scale map, we used a block penalty of 10 (hereafter referred to as the fine-scale map). A comparison of broad and fine-scale maps for a representative region of the genome is shown in Figure S2. We analyzed a total of 44,835,801 phased SNPs across the 19 mouse autosomes and the X-chromosome. From the broad-scale map, the frequency-weighted mean estimate of *ρ*/bp for the autosomes was 0.0092. This value is higher than the lower detection limit suggested by the simulations with and without switch errors (Figure 1). For the X-chromosome, the frequency-weighted mean ρ/bp was 0.0026, which is still above the lower detection limit (Figure 1). The lower SNP density on the X-chromosome (Table S3), and the smaller number of alleles available (17 compared to 20 used for the autosomes), may reduce precision.

We assessed variation in whole-chromosome recombination rates between our LD-based *castaneus* map and direct estimates of recombination rates published in earlier studies. Comparing the mean recombination rates of whole chromosomes provides us with a baseline for which we have two *a priori* expectations. First, we expect that chromosome 19, the shortest in physical length, should have the highest mean recombination rate, since at least one crossing-over event is required per meiosis per chromosome. Second, we expect that the X-chromosome, which only undergoes recombination in females, should have the lowest rate. These expectations are borne out in the results (Table 1), and are consistent with previous studies (Jensen-Seaman *et al.* 2004; Cox *et al.* 2009). We also found that frequency-weighted chromosomal recombination rates (inferred in terms of *ρ* = *4N_e_r*) were highly correlated with the direct estimates (in centimorgans per megabase pair) from Jensen-Seaman *et al.* (2004) (Pearson correlation coefficient = 0.59, *P* = 0.005) and Cox *et al.* (2009) (Pearson correlation coefficient = 0.68, *P* = 0.001). Excluding the X-chromosomes does not substantially change these correlations. These results therefore suggest that our analysis captures real variation in the rate of recombination on the scale of whole chromosomes.

### Comparison of the *M. m. castaneus* map with maps constructed using inbred lines

**Figure 2:**
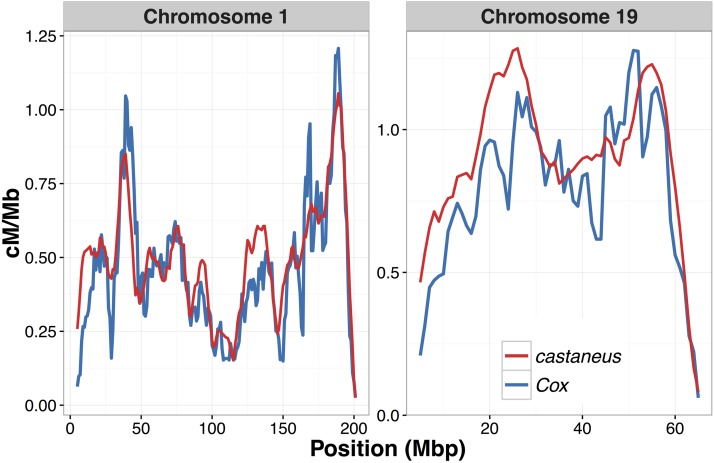
Comparison of sex-averaged recombination rates for chromosomes 1 and 19 of *M. musculus castaneus* inferred by LDhelmet (red) with rates estimated in the pedigree-based study of Cox *et al.* (2009) (blue). Recombination rates were scaled to units of centimorgans per megabase for the *castaneus* map by setting the total map length of each chromosome to the corresponding map length of Cox *et al.* (2009).

**Figure 3:**
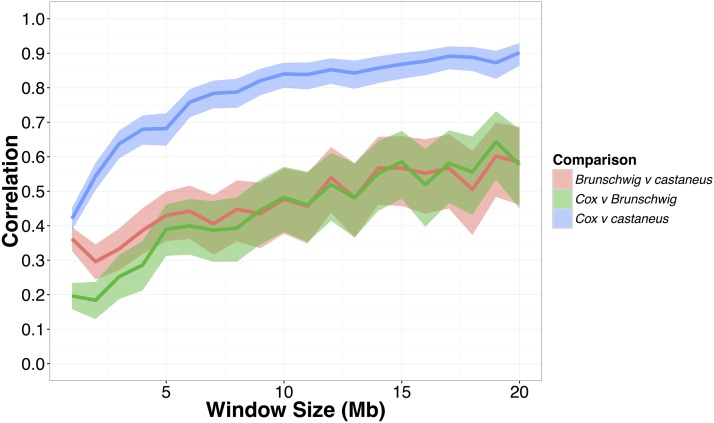
Pearson correlation coefficients between the recombination map inferred for *M. m. castaneus*, the Brunschwig *et al.* (2012) map and the Cox *et al.* (2009) map. Correlations were calculated in nonoverlapping windows of varying size across all autosomes. Confidence intervals (95%) are indicated by shading.

We then compared intrachromosomal variation in recombination rates between our broad-scale *castaneus* map and previously published maps. Figure 2 shows a comparison of recombination rates inferred from the *castaneus* and Cox maps for the longest and shortest autosomes, chromosomes 1 and 19, respectively. It is clear that the *castaneus* and Cox maps are very similar (see also Figure S3). We compared recombination rates in the *castaneus* and Cox maps in genomic intervals of various sizes, and found that correlation coefficients were >0.8 for window sizes of ≥8 Mbp (Figure 3). The correlations are smaller if chromosomes are considered separately (Figure S4). Although the correlation coefficients are generally high (Figure 3), there are several regions of the genome where the *castaneus* and Cox maps have substantially different recombination rates, for example, in the center of chromosome 9 (Figure S3). The Cox and *castaneus* maps are more similar to one another than either are to the Brunschwig map (Figure 3). This is presumably because the Brunschwig map was constructed with a relatively low SNP density and by an LD-based approach using a sample of inbred mouse strains, which violates key assumptions of the method. Population structure in the lines analyzed by Brunschwig *et al.* (2012) or the subspecies from which they were derived would elevate LD, resulting in lower chromosome-wide values of *ρ*. The average scaled recombination rate estimates differ substantially between the *castaneus* and Brunschwig maps, *i.e.*, the *castaneus* chromosomal estimates are ∼500× higher (Table 1). This is also reflected in *N_e_*, estimated on the basis of the frequency-weighted average recombination rates for each chromosome. Independent polymorphism data suggest that effective populations sizes for *M. m. castaneus* and *M. m. domesticus* are ∼100,000 and 500,000, respectively (Geraldes *et al.* 2008, 2011). Estimates of *N_e_* from the *castaneus* map are therefore in line with expectation, while those from the Brunschwig map are not (Table 1).

### Analysis of fine-scale recombination rates

To locate potential recombination hotspots in wild *M. m. castaneus*, we generated a fine-scale map, from which we identified 39,972 potential recombination hotspots. For each chromosome, there was an average of 15 hotspots per megabase pair. The total number of putative hotspots is more than twice the number identified in CAST/EiJ, an inbred strain derived from wild *M. m. castaneus* (Smagulova *et al.* 2016).

To obtain a measure of the amount of fine-scale recombination rate heterogeneity across the genome, we constructed Lorenz curves and calculated their Gini coefficients (Figure S5). The mean Gini coefficient for all chromosomes was 0.78. This estimate is very similar to that of Kaur and Rockman’s (2014) median Gini coefficient of 0.77 for chromosome 1, obtained from a high-density map of crossing-over locations in inbred mice (Paigen *et al.* 2008). The Gini coefficients calculated from our fine-scale map suggest that the distribution of recombination rates in wild and inbred mice are similarly heterogeneous. However, the Lorenz curve for the X-chromosome is clearly distinct from that of the autosomes (Figure S5), and its Gini coefficient is 0.95.

There was only a small amount of overlap between the locations of putative recombination hotspots we identified in wild *castaneus* and the locations of DSB hotspots observed in wild-derived inbred strains (Smagulova *et al.* 2016) (Table S4). As may be expected, DSB hotspots in the inbred strain derived from *M. m. castaneus* (CAST) exhibited the greatest amount of overlap with the locations of recombination hotspots identified in *M. m. castaneus*. Of all DSB hotspots in CAST, 12.2% (or 4.1% after correcting for the null expectation) overlapped with one of the putative hotspots we identified. Such a low proportion strongly suggests that, even within the *M. m. castaneus* subspecies, the locations of recombination hotspots are highly variable. The PWD strain, which was derived from wild *M. m. musculus*, exhibited the second highest amount of overlap; <1% of the DSB hotspots in each of the three strains derived from *M. m. domesticus* overlapped with putative hotspots in *M. m. castaneus*, after correcting for the number of overlaps expected to be seen by chance. Table S4 shows the overlap for each of the strains analyzed by Smagulova *et al.* (2016).

### Correlation between recombination rate and properties of protein coding genes

There is evidence of pervasive natural selection acting in protein-coding genes and conserved noncoding elements of the murid genome (Halligan *et al.* 2010, 2011, 2013). This is expected to reduce diversity at linked neutral sites via background selection and/or selective sweeps, and is therefore expected to generate a positive correlation between diversity and recombination rate, as has been observed in multiple species (Cutter and Payseur 2013).

We examined the correlation between genetic diversity and recombination rate to determine whether our map captures variation in *N_e_* across the genome. We found that the rate of recombination at autosomal protein-coding genes is significantly and positively correlated with genetic diversity of putatively neutral sites (Table 2). Furthermore, the correlation between recombination rate and neutral diversity scaled by divergence (from the rat) was both positive and significant, regardless of base context (Figure S6 and Table 2). This indicates that natural selection may have a role in reducing diversity via hitchhiking and/or background selection.

**Table 2:** Correlation coefficients between recombination rate and pairwise nucleotide diversity and divergence from the rat at fourfold degenerate sites for protein coding genes

	Correlation Coefficient	
	Non-CpG Prone Sites	All Sites
Nucleotide diversity (*π*)	0.090	0.20
Divergence from rat (*d_rat_*)	−0.038	0.062
Corrected diversity (*π/d_rat_*)	0.10	0.18

Nonparametric Kendall correlations were calculated for non-CpG prone sites and for all sites, regardless of base context. All coefficients shown are highly significant (*P* < 10^−10^).

Biased gene conversion can influence levels of between-species nucleotide substitution (Duret and Galtier 2009). GC-biased gene conversion (gcBGC), where G/C alleles are preferentially chosen as the repair template following DSBs, can generate a positive correlation between nucleotide divergence and recombination rate (Duret and Arndt 2008). Gene conversion occurs whether or not a DSB is resolved by crossing-over (Duret and Galtier 2009) and models of gcBGC predict an increase in the rates of nucleotide substitution in regions of high crossing-over (Duret and Arndt 2008). Indeed, human–chimp divergence is positively correlated with rates of crossing-over when considering all base contexts. Consistent with this, we found that fourfold site nucleotide divergence was significantly positively correlated with recombination rate for the case of all sites (Table 2). In the case of non-CpG-prone sites, however, we found only a weak negative correlation (Table 2). A recent study by Phung *et al.* (2016) found a positive correlation between human–chimp divergence and recombination rate that persisted after removing CpG-prone sites, so further study is required to analyze the effects of gene conversion on patterns of divergence in mice.

## Discussion

Our analyses suggest that the recombination landscapes of wild house mice and their laboratory counterparts are similar at broad-scales, but are dissimilar at fine-scales. Our broad-scale map captures variation in the recombination rate similar to that observed in a more traditional linkage map, both at the level of whole chromosomes and genomic windows of varying sizes. However, we found that a relatively small proportion of DSB hotspots identified in wild-derived strains (Smagulova *et al.* 2016) overlapped with putative recombination hotspots in *M. m. castaneus*. This suggests that recombination rates are highly variable within, and between, the subspecies at the kilobase scale. We discuss potential reasons for this below.

Recombination landscapes inferred using coalescent approaches, as in this study, reflect ancestral variation in recombination rates. In *M. m. castaneus*, we have shown that this ancestral variation is highly correlated with contemporary recombination rate variation in inbred mice derived from *M. m. domesticus*, suggesting that the broad-scale genetic map has not evolved substantially since the subspecies shared a common ancestor, ∼350,000 years ago (Geraldes *et al.* 2011). At a finer scale, however, there is considerable variation in the locations of recombination hotspots between the *M. musculus* subspecies. This was also observed in studies of the great-apes, which suggested that the locations of recombination hotspots have strongly diverged between species, but that broad-scale patterns are relatively conserved (Lesecque *et al.* 2014; Stevison *et al.* 2016). There are, however, several relatively large regions of the genome showing substantially different recombination rates between our *M. m. castaneus* map and the Cox map. For example, there are recombination rate peaks in *M. m. castaneus* on chromosomes 4, 5, 14, and 15, which are not present in the Cox map (Figure S3). Directly estimating recombination rates at fine scales in *M. m. castaneus* individuals could potentially reveal whether the broad-scale differences in recombination rate, mentioned above, are present in modern day populations.

The positive correlation between the *castaneus* map and the Cox map (constructed using a pedigree-based approach) is weaker for the X-chromosome than for autosomes of similar physical length (*e.g.*, chromosomes 2 and 3) (Figure S4). However, SNP density on the *M. m. castaneus* X-chromosome is substantially lower than the autosomes (Table S3). Greater physical distance between adjacent SNPs restricts the resolution of recombination rates in the coalescent-based approach. Thus, in our study, recombination rates are resolved at finer scales on the autosomes than on the X-chromosome. Additionally, we inferred recombination rates on the X-chromosome using 17 gene copies rather than the 20 used for the autosomes. Our findings are consistent, however, with the results of Dumont *et al.* (2011), who constructed linkage maps in *M. m. castaneus* and *M. m. musculus* (both by crossing with *M. m. domesticus*) using a small number of markers. In that study, the authors found multiple genomic intervals that significantly differed in genetic map distance between the two subspecies, and a disproportionate number of differences were on the X-chromosome. Thus, their results and ours suggest that the recombination landscape of the X-chromosome has evolved faster than that of the autosomes.

A recent study by Stevison *et al.* (2016) examined pairs of great ape species, and found that correlations between recombination maps (at the 1 Mbp scale) declined with genetic divergence. For example, between humans and gorillas, genetic divergence is ∼1.4%, while the Spearman-rank correlation of their respective recombination rate maps is ∼0.5. Genetic divergence between *M. m. castaneus* and *M. m. domesticus* is reported to be ∼0.5% (Geraldes *et al.* 2008), and we find a Spearman-rank correlation of 0.47 between the *castaneus* map and the Cox map, also at the 1 Mbp scale. Although this is only a single data point, it suggests that recombination rate differences may have accumulated faster relative to divergence between *M. m. castaneus* and *M. m. domesticus* than they have between great ape species. The recombination maps constructed for the great apes by Stevison *et al.* (2016) were all generated using the same methodology, which is not the case for the comparison we make between our map and that of Cox *et al.* (2009), so quantitative comparisons between the studies should be treated with caution. Performing a comparative analysis of recombination rates in the different subspecies of house mice and related mouse species (for example, *Mus caroli* and *Mus spretus*) using LD-based methods may help us understand whether the rate of evolution of the recombination landscape in wild mice is more rapid than in the great apes.

The locations of the vast majority of recombination hotspots in mice are directed by the binding of the *PRDM9* protein (Brick *et al.* 2012), and there are unique landscapes of DSB hotspots associated with the different *PRDM9* alleles present in different wild-derived inbred strains (Smagulova *et al.* 2016). However, in natural populations there is a great diversity of *PRDM9* alleles in each of the *M. musculus* subspecies (Kono *et al.* 2014), therefore the binding motif will vary, causing different suites of hotspot locations. Thus, the DSB hotspot maps obtained by Smagulova *et al.* (2016) likely represent a fraction of the diversity of hotspot locations in wild *M. musculus* populations. Indeed, we found that only 12% of the DSB hotspots reported for CAST/EiJ by Smagulova *et al.* (2016) overlapped with hotspots we inferred for *M. m. castaneus* (Table S4). However, the mean Gini coefficient we estimated for *M. m. castaneus* was almost identical to the value obtained by Kaur and Rockman (2014) from crossing-over data of *M. musculus*. This similarity suggests that, while the locations of hotspots may differ, the distribution of recombination rates is similarly heterogeneous in wild and inbred mice.

The *castaneus* map constructed in this study appears to be more similar to the Cox map than the Brunschwig map (Figure 3). There are number of potential reasons for this. First, we used a much larger number of markers to resolve recombination rates than Brunschwig *et al.* (2012). Second, it seems probable that population structure within, and between, the inbred and wild-derived lines studied by Brunschwig *et al.* (2012) could have resulted in biased estimates of the recombination rate. The Brunschwig map does, however, capture true variation in the recombination rate, since their map is also highly correlated with the Cox map (Pearson correlation >0.4) for all genomic windows >8 Mbp (Figure 3). Indeed, Brunschwig *et al.* (2012) showed by simulation that hotspots are detectable by analysis of inbred lines, and validated their hotspots against the locations of those observed in crosses among classical strains of *M. m. domesticus* (Smagulova *et al.* 2011). This suggests that while estimates of the recombination rate in the Brunschwig *et al.* (2012) map may have been downwardly biased by population structure (see above), variation in the rate and locations of hotspots were still accurately detected.

By simulating the effect of switch errors on estimates of the recombination rate, we inferred the range over which *ρ*/bp is accurately estimated. Switch errors appear identical to legitimate crossing-over events, and, if they are randomly distributed along chromosomes, a specific rate of error will resemble a constant rate of crossing-over. The rate of switch error will then determine a detection threshold below which recombination cannot be accurately inferred. We investigated this detection threshold by introducing switch errors, at random, into simulated data at the rate we estimated using the X-chromosome. We found that, in the presence of switch errors, LDhelmet consistently overestimates the recombination rate when the true value is below 2 × 10^−3^ *ρ*/bp (Figure 1 and Figure S1). This highlights a possible source of bias affecting LD-based recombination mapping studies that use inferred haplotypes, and suggests that error in phase inference needs to be carefully considered.

We obtained an estimate of the switch error rate, using a novel approach that took advantage of the hemizygous sex chromosomes of males. This allowed us to assess the extent by which switch errors affected our ability to infer recombination rates. Our inferred switch error rate may not fully represent that of the autosomes, however, because multiple factors influence the ability to phase variants (*i.e.*, LD, SNP density, sample size, depth of coverage, and read length), and some of these factors differ between the X-chromosome and the autosomes. The sex-averaged recombination rate for the X-chromosome is expected to be three-quarters that of the autosomes, so it will likely have elevated LD, and thus there will be higher power to infer phase. In contrast, X-linked nucleotide diversity in *M. m. castaneus* is approximately one-half that of the autosomes (Kousathanas *et al.* 2014), so there would be a higher number of phase informative reads on the autosomes. While it is difficult to assess whether the switch error rates we estimated from the X-chromosome will be similar to those on the autosomes, the analysis allowed us to explore the effects of different SNP filters on the error rate.

Consistent with studies in a variety of organisms (Cutter and Payseur 2013), we found a positive correlation between genetic diversity at putatively neutral sites and the rate of recombination. Both unscaled nucleotide diversity and diversity divided by divergence between mouse and rat, a proxy for the mutation rate, are positively correlated with the recombination rate (Table 2). Cai *et al.* (2009) found evidence suggesting that recombination may be mutagenic, although insufficient to account for the correlations they observed. The Kendall correlation between *π/d_rat_* and recombination rate is 0.20 for all fourfold sites (Table 2), which is similar in magnitude to the corresponding value of 0.09 reported by Cai *et al.* (2009) in humans. The correlations we report may be downwardly biased, however, because switch errors may result in inflated recombination rates for genomic regions where the recombination rate is low (see above). Genes that have recombination rates lower than the detection limit set by the switch error rate may be reported as having inflated *ρ*/bp (Figure 1 and Figure S1), and this would have the effect of reducing correlation statistics. It is difficult to assess the extent of this bias, however, and, in any case, the correlations we observed between diversity and recombination suggest that our recombination map does indeed capture real variation in *N_e_* across the genome. This indicates that a recombination-mediated process influences levels of genetic diversity. Previously, Halligan *et al.* (2013) showed that there are reductions in nucleotide diversity surrounding protein coding exons in *M. m. castaneus*, characteristic of natural selection acting within exons reducing diversity at linked sites. Their results and ours suggest pervasive natural selection in the *M. m. castaneus* genome. In contrast, a previous study in wild mice found that, while *M. m. musculus* exhibited a significant correlation between diversity and recombination, the relationship was nonsignificant for both *M. m. castaneus* and *M. m. domesticus* (Geraldes *et al.* 2011). This study analyzed only 27 loci, so was perhaps underpowered to detect a relatively weak correlation. It should be noted, however, that the measure of recombination rate we used (*ρ*/bp) and neutral genetic diversity are both functions of the effective population size, so the positive correlation we detected could be partly driven by random fluctuations of *N_e_* across the genome.

Furthering our understanding of the evolution of the recombination landscape in house mice would be helped by comparing fine-scale rates in the different subspecies. In this study, we have assumed that inbred lines derived from *M. m. domesticus* reflect natural variation in recombination rates in that subspecies, though this is not necessarily the case. Directly comparing natural population samples of the different subspecies may help reconcile several potentially conflicting results. For example, the hotspots we detected in our study show more overlap with *M. m. musculus* than with *M. m. domesticus*, based on the DSB hotspots reported by Smagulova *et al.* (2016). However, overall rates of crossing-over in male *M. m. musculus* are higher than in either *M. m. castaneus* or *M. m. domesticus* (Dumont and Payseur 2011). Additionally, there is evidence of recombination rate modifiers of large effect segregating within *M. m. musculus* populations (Dumont *et al.* 2011). So, although overall rates of crossing-over in *M. m. musculus* are higher than in the other species, its recombination landscape may be more similar to *M. m. castaneus* than to *M. m. domesticus*. A broad survey comparing recombination rate landscapes in the different subspecies of mice would most efficiently be performed using LD-based approaches.

In conclusion, we find that sex-averaged estimates of the ancestral recombination landscape for *M. m. castaneus* are highly correlated with contemporary estimates of the recombination rate observed in crosses of inbred lines that predominantly reflect *M. m. domesticus* (Cox *et al.* 2009). It has previously been demonstrated that the turnover of hotspots has led to rapid evolution of fine-scale rates of recombination in the *M. musculus* subspecies complex (Smagulova *et al.* 2016), and our results suggest that even within *M. m. castaneus* hotspot locations are variable. On a broad scale, however, our results suggest that the recombination landscape is very strongly conserved between *M. m. castaneus* and *M. m. domesticus* at least. In addition, our estimate of the switch-error rate implies that phasing errors lead to upwardly biased estimates of the recombination rate when the true rate is low. This is a source of bias that should be assessed in future studies. Finally, we showed that the variation in recombination rate is positively correlated with genetic diversity, suggesting that natural selection reduces diversity at linked sites across the *M. m. castaneus* genome, consistent with the findings of Halligan *et al.* (2013).

## Supplementary Material

Supplemental material is available online at www.genetics.org/lookup/suppl/doi:10.1534/genetics.117.300063/-/DC1.
