# Supplementary figures and images for: The Recombination Landscape in Wild House Mice Inferred Using Population Genomic Data
Source: Genetics. 2017 Jul 26;207(1):297–309. doi: 10.1534/genetics.117.300063 (PMC5586380; doi:10.1534/genetics.117.300063)

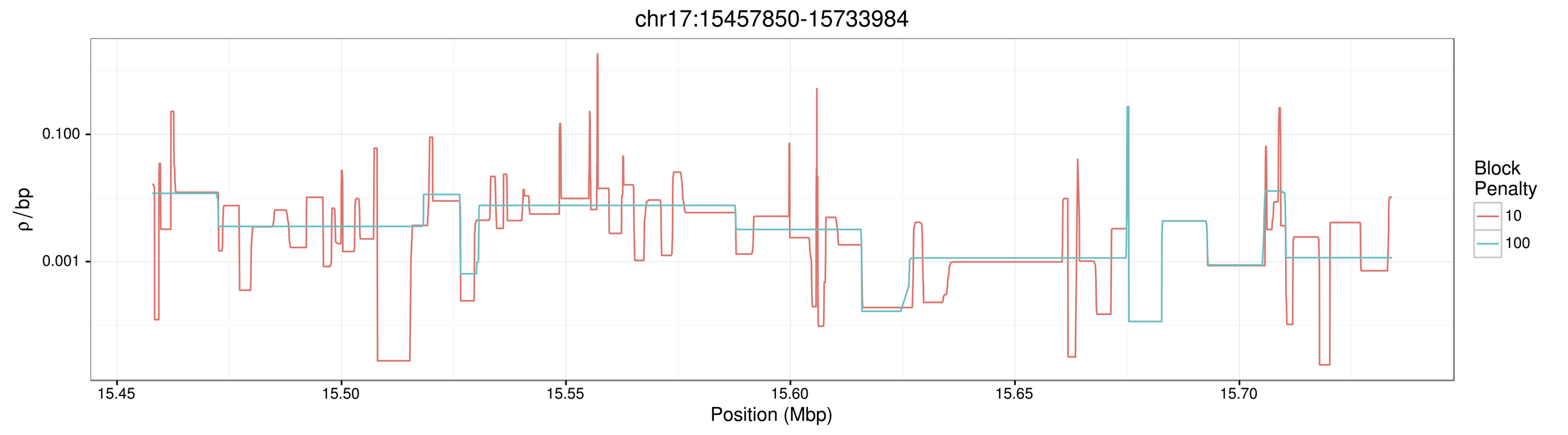

Supplement: Supplementary file 2 [file 297FigureS2.jpg]

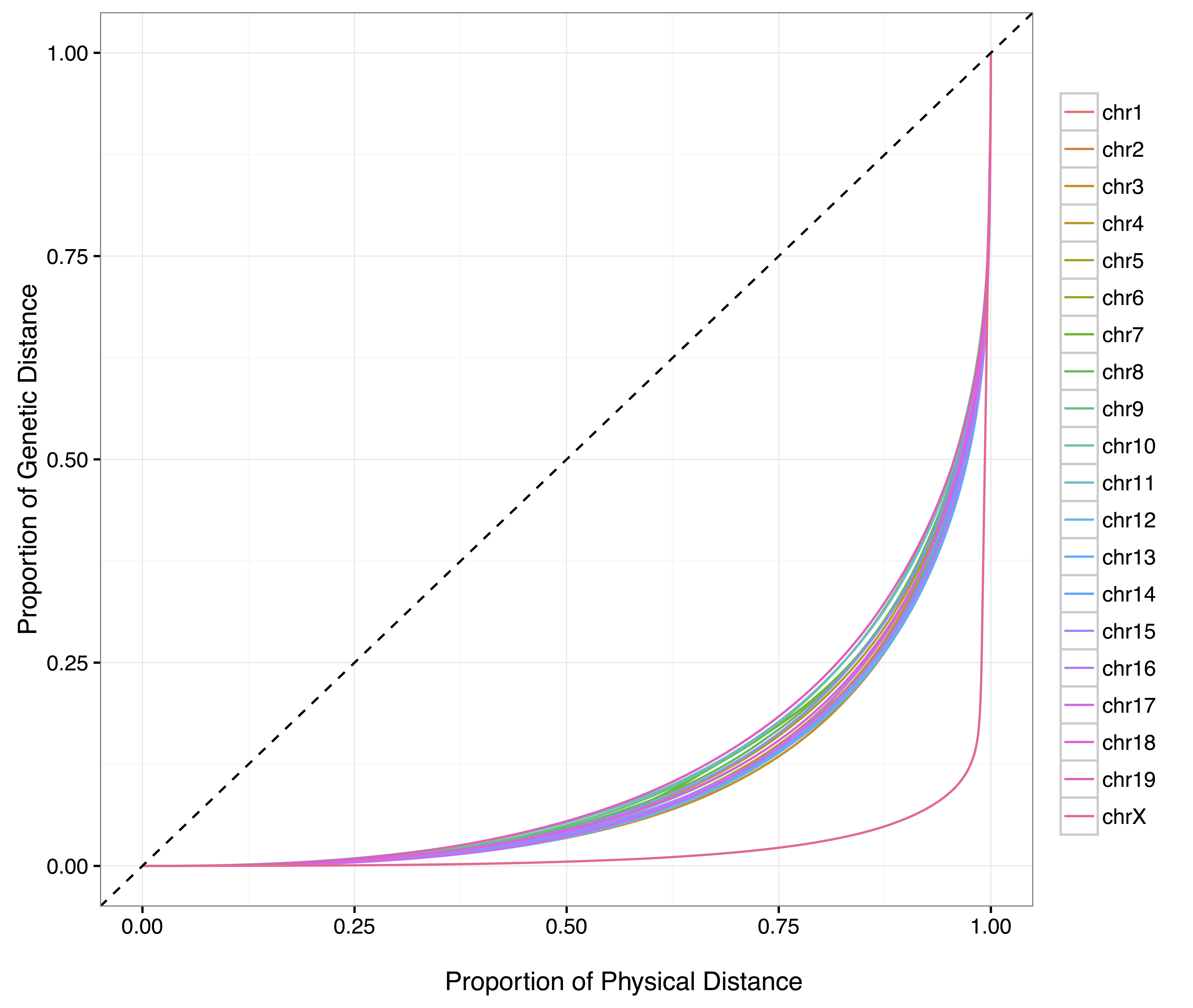

Supplement: Supplementary file 5 [file 297FigureS5.jpg]
