# Supplementary material for: The Recombination Landscape in Wild House Mice Inferred Using Population Genomic Data
Source: Genetics. 2017 Jul 26;207(1):297–309. doi: 10.1534/genetics.117.300063 (PMC5586380; doi:10.1534/genetics.117.300063)
Supplement: Supplementary file 7 [file 297TableS1.docx]

**Table S1**

The effect of different filters on the frequency of switch errors in the haplotypes inferred based on the three pseudo-females (H40, H46 and H62). The values in the switch errors column are the raw numbers of switch errors and the total number of heterozygous SNPs on the X-chromosome. Variants with Quality (QUAL) <30 were excluded for all filter sets.

| **Filter Set** | **HWE^*^** | **Min DP^†^** | **Max DP** | **Min GQ^‡^** | **Switch Errors** | | | **Switch Error**  **Rate** |
| --- | --- | --- | --- | --- | --- | --- | --- | --- |
|  |  |  |  |  | ***H40*** | ***H46*** | ***H62*** |  |
| **1** | - | - | - | - | 5148 /  409486 | 4819 /  407422 | 5020 /  394778 | 0.0124 |
| **2** | <0.0002 | 10 | - | 15 | 1690 /  338592 | 1451 /  334111 | 1452 /  324199 | 0.0046 |
| **3** | <0.0002 | 10 | 100 | 5 | 2460 /  341744 | 2066 /  339508 | 2536 /  328998 | 0.0070 |
| **4** | <0.0002 | - | - | 40 | 523 /  288471 | 444 /  286636 | 550 /  281266 | 0.0018 |

*^*^HWE refers to the p-value for the Samtools Hardy-Weinberg equilibrium test below which variants were excluded.*

^†^*Depth of coverage per individual.*

^‡^*Per individual genotype quality score.*
