# Supplementary material for: The Recombination Landscape in Wild House Mice Inferred Using Population Genomic Data
Source: Genetics. 2017 Jul 26;207(1):297–309. doi: 10.1534/genetics.117.300063 (PMC5586380; doi:10.1534/genetics.117.300063)
Supplement: Supplementary file 8 [file 297TableS2.docx]

**Table S2**

The normalized mutation rate matrix and stationary distribution of base frequencies estimated with two outgroups, *M. famulus* and *R. norvegicus*, using the method described by Chan *et al.* (2012).

|  | A | C | G | T |
| --- | --- | --- | --- | --- |
| A | 0.48 | 0.09 | 0.32 | 0.11 |
| C | 0.19 | 0.00 | 0.12 | 0.69 |
| G | 0.69 | 0.12 | 0.00 | 0.19 |
| T | 0.11 | 0.32 | 0.08 | 0.48 |
| Stationary  Distribution | 0.34 | 0.16 | 0.16 | 0.34 |
