# Supplementary material for: The Recombination Landscape in Wild House Mice Inferred Using Population Genomic Data
Source: Genetics. 2017 Jul 26;207(1):297–309. doi: 10.1534/genetics.117.300063 (PMC5586380; doi:10.1534/genetics.117.300063)
Supplement: Supplementary file 9 [file 297TableS3.docx]

**Table S3**

The total number of SNPs in the dataset, and the number of SNPs after applying filters.

|  |  | **# SNPs** | |
| --- | --- | --- | --- |
| **Chromosome** | **Physical Size (Mbp)** | **Raw** | **Filtered** |
| 1 | 197.2 | 6,250,153 | 3,557,581 |
| 2 | 181.7 | 5,420,000 | 3,095,049 |
| 3 | 159.6 | 5,207,849 | 2,961,039 |
| 4 | 155.6 | 4,916,193 | 2,655,529 |
| 5 | 152.5 | 4,786,546 | 2,639,326 |
| 6 | 149.5 | 4,831,712 | 2,658,278 |
| 7 | 152.5 | 4,296,986 | 2,266,748 |
| 8 | 131.7 | 4,089,400 | 2,309,811 |
| 9 | 124.1 | 3,871,695 | 2,221,982 |
| 10 | 130.0 | 4,323,747 | 2,440,209 |
| 11 | 121.8 | 3,744,895 | 2,100,852 |
| 12 | 121.3 | 3,674,871 | 2,036,520 |
| 13 | 120.3 | 3,760,538 | 2,137,776 |
| 14 | 125.2 | 3,874,312 | 2,164,901 |
| 15 | 103.5 | 3,333,449 | 1,877,022 |
| 16 | 98.3 | 3,193,551 | 1,822,476 |
| 17 | 95.3 | 3,111,409 | 1,627,303 |
| 18 | 90.8 | 2,926,381 | 1,692,050 |
| 19 | 61.3 | 1,949,809 | 1,101,783 |
| X | 166.7 | 2,535,365 | 1,469,566 |
|  |  |  |  |
| Total |  | 80,098,861 | 44,835,801 |
