# Supplementary material for: The Recombination Landscape in Wild House Mice Inferred Using Population Genomic Data
Source: Genetics. 2017 Jul 26;207(1):297–309. doi: 10.1534/genetics.117.300063 (PMC5586380; doi:10.1534/genetics.117.300063)
Supplement: Supplementary file 10 [file 297TableS4.docx]

**Table S4**

The overlap between the hotspots we identified in *M. m. castaneus* and the locations of DSB hotspots in wild-derived strains obtained by Smagulova *et al.* (2016). The corrected overlap (%) is the number of overlapping hotspots, above the null expectation, divided by the total.

| **Strain ID** | **Sub-species** | **# DSB Hotspots** | **# Overlaps** | **% Overlap - Uncorrected** | **Null Expectation** | **% Overlap - Corrected** |
| --- | --- | --- | --- | --- | --- | --- |
| 13R | *domesticus* | 14,744 | 1,202 | 8.2 | 1,169 | 0.2 |
| B6 | *domesticus* | 19,455 | 1,533 | 7.9 | 1,505 | 0.1 |
| C3H | *domesticus* | 14,635 | 1,399 | 9.6 | 1,308 | 0.6 |
| CAST | *castaneus* | 15,061 | 1,831 | 12.2 | 1,221 | 4.1 |
| MOL | *molossinus* | 15,718 | 1,559 | 9.9 | 1,351 | 1.3 |
| PWD | *musculus* | 14,483 | 1,569 | 10.8 | 1,205 | 2.5 |
